# Interplay between Polaritonic and Molecular Trap States

**DOI:** 10.1021/acs.jpcc.2c01239

**Published:** 2022-05-03

**Authors:** Jürgen Mony, Yi Yu, Clara Schäfer, Suman Mallick, Khushbu Kushwaha, Karl Börjesson

**Affiliations:** Department of Chemistry and Molecular Biology, University of Gothenburg, Kemigården 4, Gothenburg 41296, Sweden

## Abstract

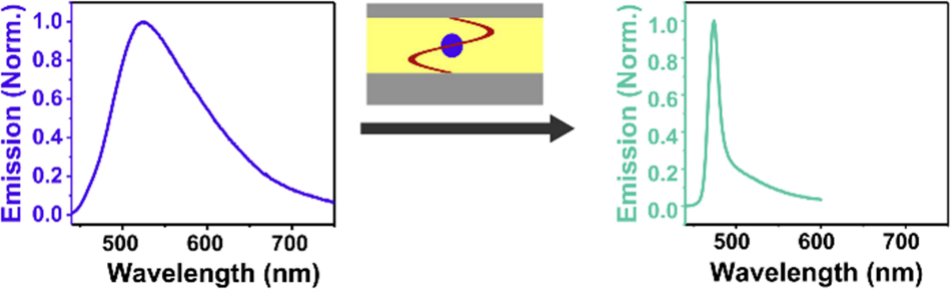

Strong exciton–photon coupling exhibits the possibility to modify the photophysical properties of organic molecules. This is due to the introduction of hybrid light–matter states, called polaritons, which have unique physical and optical properties. Those strongly coupled systems provide altered excited-state dynamics in comparison to the bare molecule case. In this study, we investigate the interplay between polaritonic and molecular trap states, such as excimers. The molecules used in this study show either prompt or delayed emission from trap states. For both cases, a clear dependency on the exciton–photon energy tuning was observed. Polaritonic emission gradually increased with a concurrent removal of aggregation-induced emission when the systems were tuned toward lower energies. For prompt emission, it is not clear whether the experimental results are best explained by a predominant relaxation toward the lower polariton after excitation or by a direct excimer to polariton transition. However, for the delayed emission case, trap states are formed on the initially formed triplet manifold, making it evident that an excimer-to-polariton transition has occurred. These results unveil the possibility to control the trap state population by creating a strongly coupled system, which may form a mitigation strategy to counteract detrimental trap states in photonic applications.

## Introduction

Organic chromophores with extensive conjugation generally exhibit a large, rigid π-core. Because of their planarity and high polarizability, they have a tendency to stack together through strong π–π interactions. Depending on subtleties in intermolecular distances and angles, altered photophysics of formed aggregates can be classified into H- and J-aggregates,^1^ excimers,^2−4^ charge transfer states,^5^ and aggregated trap states.^6^ The dimerization of molecules in the excited state, called excimers, generally leads to a quenching of the photoluminescence accompanied by a spectral red-shift and broadening.^2,7^ Therefore, their applicability in, for instance, triplet–triplet annihilation upconversion^8^ and light-emitting diodes^9^ is limited. A common synthetic approach to suppress the aggregation and circumvent the changes in optical properties is the introduction of bulky side chains into the molecular structure.^10−12^ Another method is entropic mixing, where chromophores with different alkyl side chains are blended together, resulting in a stronger glass-forming capability.^13−15^ This attempt of reducing the π–π-interactions and therefore the aggregation is favorable for applications where the molecular size and weight play a major role. However, it is sometimes difficult to completely remove the formation of detrimental aggregation, and in such cases, a higher degree of control of excited-state relaxation pathways is crucial to prevent photon emission losses.

Strong exciton–photon coupling enables the modification of photochemical and photophysical properties of molecules without any synthetic modification.^16−19^ A strong coupling regime is achieved when the interaction between light and matter is to an extent that their states cannot be treated as linearly independent entities anymore. This leads to the formation of hybrid light–matter energy states, called polaritons, which are separated in energy by the Rabi splitting.^20,21^ Being partially light and matter, these new hybrid states inherit properties of both parts and show unique optical properties. Polariton emission is both angle-dependent and shows an extremely sharp envelope,^22^ beneficial in a number of photonic applications like organic light-emitting diodes with high color purity. Under resonance conditions between the molecular transition and an optical cavity mode, the polaritons have equal photonic and excitonic contributions. The ratio between the photonic and excitonic contributions can be adjusted by shifting the energy of the cavity resonance. For a cavity resonance below the excitonic energy, the photonic contribution to the lower polariton is increased. This is generally referred to as red-detuning. Blue-detuning is the opposite process, where the cavity energy is higher than the exciton energy, resulting in a lower photonic component to the lower polariton. The energy of the polaritons is also affected following the energy of the cavity mode. The energetics and composition of polaritons can thus be simply tuned by experimentally controlling the cavity energy.

The dynamics of the relaxation to the lower polariton have been extensively investigated.^23−31^ The relaxation rate is limited by the so-called dark states, which form a reservoir of states with no or very small photonic contribution at the energy of the molecular transition. The existence of the dark states is indicated by their angle-independent emission lifetime being on the timescale of the bare molecule.^32,33^ A Stokes shift dependence of the relaxation pathway has been shown; a molecule exhibiting a large Stokes shift relaxes by radiative pumping to the lower polariton, whereas a J-aggregate with a negligible Stokes shift relaxes via vibrationally assisted scattering.^27^ Furthermore, the impact of polaritons on delayed emission,^34^ emission quantum yield,^35^ and energy transfer^36−39^ and the direct interaction between molecule’s triplet state^40−42^ and triplet–triplet annihilation^34,43,44^ to polaritons have been studied. The exchange of energy between polaritons and molecular states shows a cavity tuning dependency.^41,44^ For photochemical reactions, the suppression of photobleaching^45^ and the reduction of photoisomerization efficiency^46^ when exciting the lower polariton have been demonstrated. These processes also exhibit a cavity tuning dependency. The more red-detuned the systems are, the higher is the suppression of the photobleaching and photoisomerization processes. However, it is unclear if this effect comes from the cavity tuning that changes the energetics or the value of the excitonic contribution of the lower polariton.

Here, we demonstrate that the relaxation pathways toward trap states can be modified within the strong exciton–photon coupling regime. By different tuning, emission from aggregated states can be suppressed in favor of polaritonic emission. This observation is shown for both prompt and delayed emission. Furthermore, emission from aggregated states is even more decreased when exciting the lower polaritonic state. This effect is enhanced in strongly red-detuned cavities, having both a lower energy of the polariton and a higher photonic contribution to it. This work spreads light to the subtle interactions between polaritonic and trap-states, which will aid in the understanding of exciton–polariton dynamics.

## Methods

### Sample Preparation

Cavities and the bare films were all prepared on clean glass substrates. For the samples containing 1-ethyl-perylene and isopropyl-BODIPY, a solution of the dye and polystyrene with a mass ratio of 1:1 was prepared in toluene. The final concentration for both dyes in the solution was 22 mg/mL. The bare films were made by spincoating (Laurell Technologies WS-650) the solutions on glass substrates. For the cavities, an Ag mirror (100 nm) was deposited by vacuum sputtering deposition (HEX, Korvus Technologies) on the glass substrate. This was followed by spincoating the dye/polystyrene solution on top of the mirror, and finally the optical cavity was sealed by sputtering a second Ag mirror on top (25 nm). The thickness of the optical cavity was changed by varying the rotational speed during the spincoating process (900–1700 rpm). The DABNA-2 samples were prepared using the same steps with the difference that the top Ag mirror had a thickness of 40 nm and the DABNA-2 layer was spincoated from a 20 mg/mL solution without any polymer (∼2500 rpm).

The cavities containing perylenetetracarboxylic dianhydride (PTCDA) were produced in a different way. Both Ag mirrors were prepared as described before, but the PTCDA (60 nm) was evaporated using a molecular evaporator (HEX, Korvus Technologies). To avoid contact between the pristine PTCDA film and the Ag mirrors, a PVA containing water solution (2 mg/mL) was spincoated on the bottom mirror and on the PTCDA film after the evaporation process.

### Optical Characterization

All optical measurements were carried out at room temperature. A spectrophotometer (LAMBDA 950, PerkinElmer) was used to measure the absorption spectra of the bare films and the reflectance of the cavities. The dispersive reflectance of the cavities was measured through the thinner top mirror using a universal reflectance accessory (PerkinElmer). The steady-state emission and fluorescence lifetimes were measured using a spectrofluorometer (FLS1000, Edinburgh Instruments). For the emission measurement, two liquid light guides were connected to the spectrofluorometer. The excitation light from the first light guide was collimated on the sample, and the emission was captured by the second light guide at an adjustable angle. Delayed emission spectra were recorded on an Edinburgh Instrument LP 980 spectrometer equipped with an ICCD (Andor). A Spectra-Physics Nd:YAG laser (pulse width ∼7 ns) coupled to a Spectra-Physics primoscan optical parametric oscillator was used as the pump source.

## Results and Discussion

### Introduction of the Molecules Used

**Figure 1:**
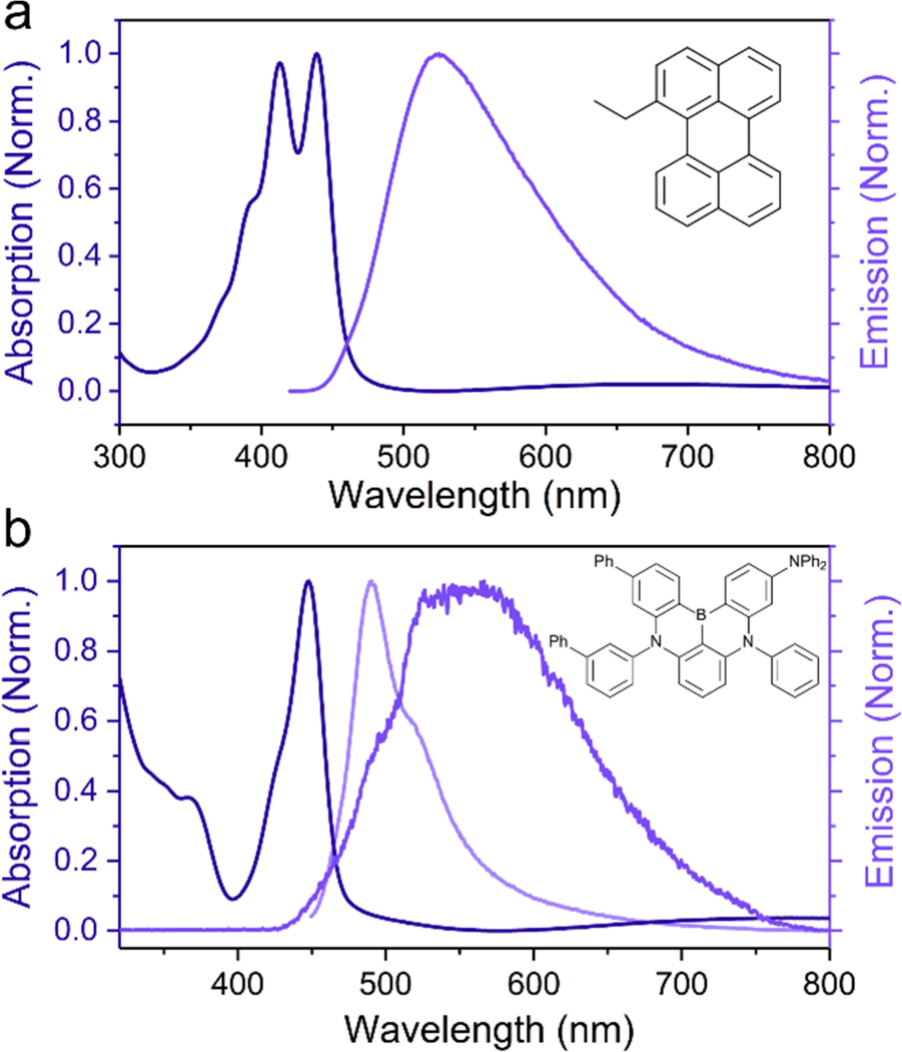
(a) Absorption and emission spectra of 1-ethyl-perylene in a polystyrene matrix and (b) absorption (dark blue), prompt emission (light blue), and delayed emission spectra (blue) of a neat DABNA-2 film at room temperature.

To study the dynamics of aggregated states in the strong coupling regime, the molecules, incorporated into the system, need to fulfill a few requirements. A quite obvious demand is that the molecules exhibit an aggregated emissive state. In addition to that, the S_1_ ← S_0_ transition dipole moment must be large enough to enable entrance into the strong coupling regime. Furthermore, the molecule must be processable into films of an appropriate thickness as to generate a resonant cavity mode. Many molecules fulfill these requirements, and the influence of the lower polariton on the photophysics of two different classes of emissive molecules will be discussed here. The first class represents the traditional organic dye, and here we chose 1-ethyl-perylene because of its tendency to aggregate at higher concentrations in a polystyrene matrix. The absorption spectrum at room temperature shows two sharp and narrow peaks at 439 and 413 nm, which represent different vibronic transitions (Figure 1a). However, the emission spectrum only consists of a broad band with a maximum at 520 nm, which is due to excimer emission.^13,47^ In addition to 1-ethyl-perylene, 3,5-isopropyl-BODIPY and PTCDA were used to further support the observations and to generalize that the results are applicable for other molecules having the tendency to form aggregated states. Their results are displayed in the Supporting Information.

The second class of molecules can undergo the process of thermally activated delayed fluorescence (TADF), represented here by DABNA-2 (Figure 1b). This class of compounds can in its triplet excited-state thermally populate the higher in energy singlet excited state via reverse intersystem crossing (RISC). This process is on a microsecond-millisecond timescale, being much longer than the fluorescence lifetimes of organic dyes, which are on a timescale of a few nanosecond. The absorption and prompt emission spectra of DABNA-2 in a neat film at room temperature show sharp and narrow transitions at 448 and 490 nm, respectively. However, the delayed emission is broad and structureless with a maximum around 600 nm. The discrepancy between prompt and delayed emission can be explained by the lifetime of the triplet state being long enough for the excited state energy to diffuse around until being caught at a trap site. Such a trap then functions as an energy sink at both the singlet and triplet manifold, and RISC then occurs between aggregation-induced trap sites. The fact that excimers on the triplet surface can be converted to excimers on the singlet surface has previously been observed when perylene undergoes triplet–triplet annihilation.^4,8^ Thus, both molecular classes exhibit emission from aggregated states in different ways; therefore they are suitable to study the behavior of aggregated states in the strong coupling regime.

### Introducing the Strongly Coupled Systems

**Figure 2:**
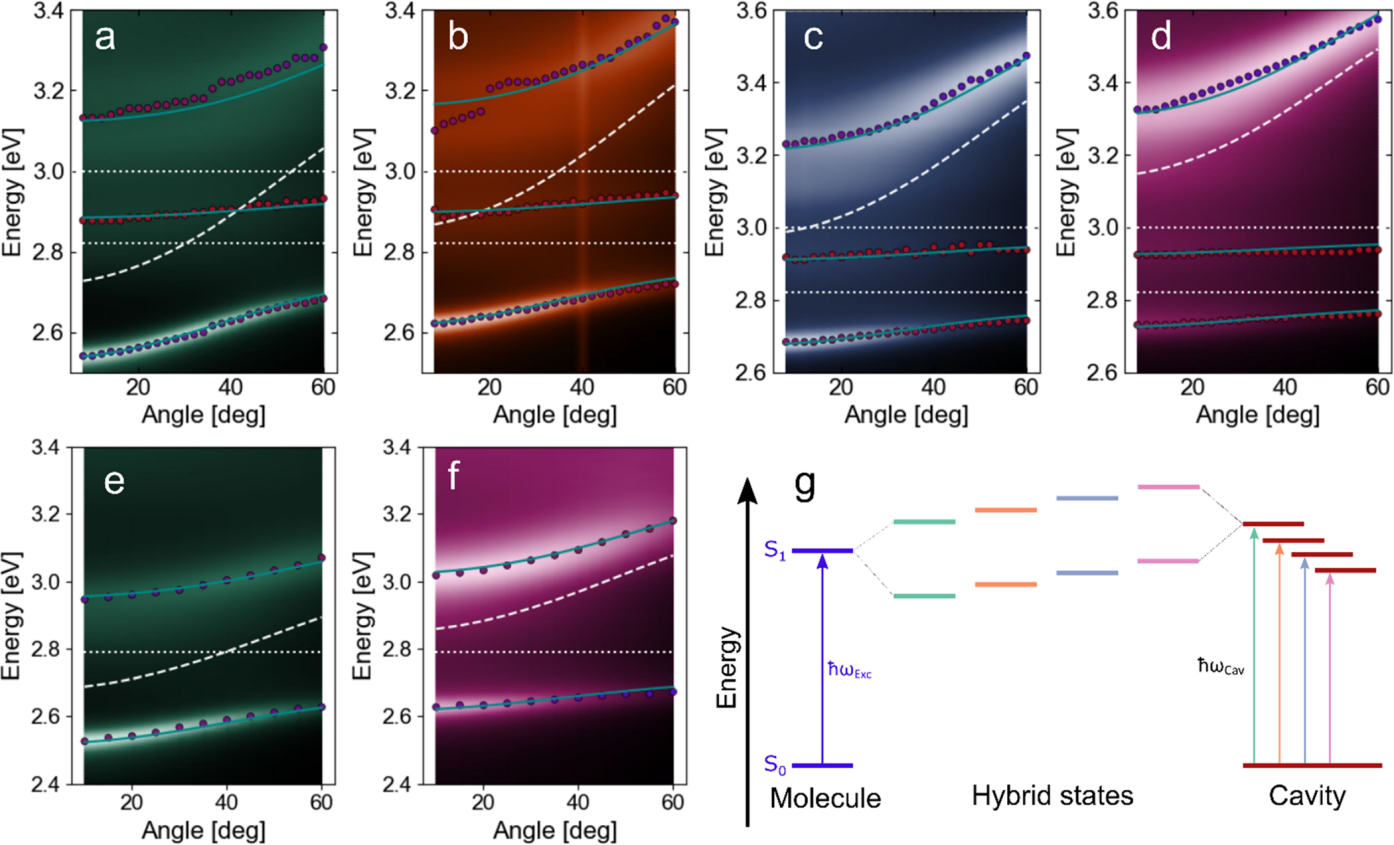
Dispersion plots for the 1-ethyl-perylene cavities (a–d) and the DABNA-2 cavities (e, f) having different tuning. The dots represent the measured minima in the reflectivity, the teal line is the fit obtained by the coupled harmonic oscillator model, the white dashed line is the cavity resonance energy, and the white dotted lines are the molecular transition energies. (g) Energy sketch of the differently detuned cavities.

The previously mentioned molecules were embedded into Fabry–Pérot cavities to enhance the light–matter interaction. The resonance of the electromagnetic field is determined by the thickness of the optical cavity, and the cavity thickness was thus used as a tuning parameter. To reach the strong exciton–photon coupling regime, the optical resonance of the cavity needs to match the exciton transition energy, and their interaction needs to be strong enough that they cannot be treated as separate entities anymore. Because of the strong interaction, the former energy states split up into new energy branches, called polaritons, separated in energy by the Rabi splitting ℏΩ_R_. These hybrid light–matter quasiparticles inherit a dispersive behavior from the photonic component. Angle-resolved reflectivity was measured on four cavities containing 1-ethyl-perylene and two cavities containing DABNA-2 (Figure 2a–f). The difference between individual cavities was their thickness, resulting in small energetic mismatches between the photonic transition and the transition maximum of the molecule (Figure 2g). Under resonance conditions, the excitonic and photonic contributions to the lower polariton are equal. For cavity resonances below the transition maximum of the molecule, the photonic contribution to the lower polariton increases, but the excitonic contribution decreases (and vice versa). In addition to the exciton–photon contribution, tuning affects the energy of the polaritons in a way that the spectral overlap between the excitonic and polaritonic states alters, which in turn affects excitonic–polaritonic exchange rates.^46^

Dispersion plots for the cavities containing 1-ethyl-perylene show a splitting into three different polaritonic states, namely, the lower, middle, and upper polaritons because of the two vibronic transitions of the molecule. All cavities display a characteristic anticrossing behavior, meaning that the polaritonic states approach the excitonic energies with increasing angle but never cross them. The results can be fitted to a three coupled harmonic oscillator model, containing one cavity and two excitonic transitions^48^
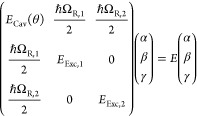
1where *E*_Cav_(θ) is the angle-dependent photon energy, *E*_Exc,1_ and *E*_Exc,2_ are the energies for the first and second vibronic transitions, and ℏΩ_R,1_ and ℏΩ_R,2_ are the corresponding Rabi splittings. The Hopfield coefficients |α|^2^, |β|^2^, and |γ|^2^ represent the photonic and excitonic contribution to the corresponding polaritons. The Rabi splittings for the 1-ethyl-perylene cavities are all in the range of 332–348 meV for the first transition and 370–384 meV for the second transition. These results indicate that the system is in the strong coupling regime because in all cases the Rabi splitting is greater than the estimated full width at half maximum (FWHM) of the first (140 meV) and second (196 meV) vibronic transition. The photonic contribution to the lower polariton spans from 0.65 for the most red-detuned cavity over 0.50 and 0.37 to 0.22 for the most blue-detuned cavity at an angle of 0 degree (Figure 2a–d). Tables S1–S4 summarize all fitting parameters for each cavity.

The dispersion plots for the cavities containing DABNA-2 also show a clear anticrossing behavior (Figure 2f,g). However, because DABNA-2 only contains one spectrally resolved vibronic transition, the system splits into two polaritonic branches, the upper and lower polaritons. The fitting model was therefore reduced to only contain one excitonic transition. The Rabi splitting for both cavities was around 400–420 meV, which is considerably larger than the FWHM of the molecular transition (∼190 meV). This indicated that the coupling strength is greater than the dissipation energy, and therefore, the system is in the strong coupling regime. The photonic contribution to the lower polariton was 0.63 and 0.43 for the two cavities.

To summarize, two classes of dyes were placed into optical microcavities, and the strong exciton photon coupling regime was reached (two other molecules are only discussed in the Supporting Information but corroborate the results). Both molecules show emission from aggregated states in bare films. However, 1-ethyl-perylene displays it in the prompt emission spectrum, whereas DABNA-2 shows it in the delayed emission spectrum. For both molecules, cavities with varying thicknesses were produced, allowing the study of tuning dependency.

### Prompt Emission Characteristics

**Figure 3:**
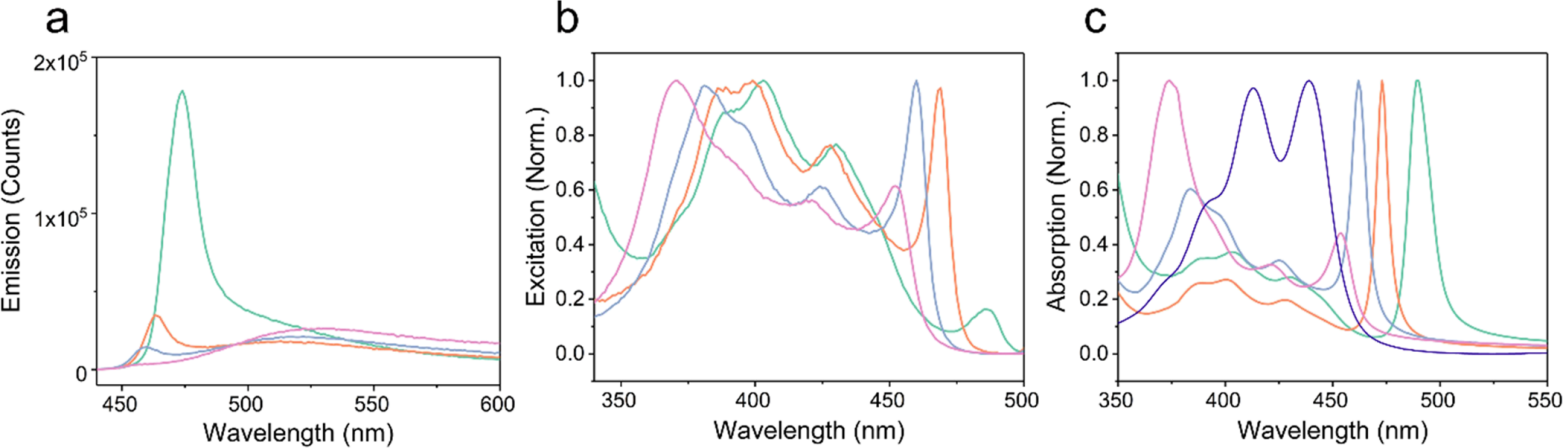
(a) Emission spectra of the different cavities containing 1-ethyl-perylene at an emission angle of 50 degree. (b) Excitation spectra for all cavities when recording the emission at 520 nm. (c) Absorption spectra for all cavities compared to the absorption of the bare film (dark blue).

The impact of strong coupling on the photophysics of prompt emission was determined with the 1-ethyl-perylene cavities. By exciting the upper polariton, the emission spectra show a clear tuning dependency (Figure 3a). In general, the excimer emission is low in the cavities, but the polariton emission exceeds the bare film emission at its maximum using the same measurement setup. This is also evident in PTCDA cavities (Figure S2). The molecular-like emission coincides with the polaritonic emission. Therefore, the emission lifetimes were determined at the molecular/polaritonic and aggregated emission wavelength to see if the dynamics are perturbed by the strong coupling regime. The decay lifetimes are at the same level for both the molecular/polaritonic and the aggregated emission wavelengths (Figure S3). The red-detuned cavity shows the most intense polaritonic emission, which gradually decreases when blue-detuning the system. For the excimer emission, the behavior was vice versa, and the most blue-detuned cavity showed the most intense excimer emission. Because the mirror thicknesses are the same throughout all the cavities, it cannot be argued that changes in the excimer emission are due to differences in mirror quality. Furthermore, the observed trend of the excimer emission cannot be reproduced by the bare film emission filtered by the reflectance spectra of the cavities (Figure S4). Therefore, the higher excimer emission must be related to the different tuning of the cavities. Furthermore, from an outcoupling perspective from the optical cavity, an enhancement of excimer emission with increasing blue-detuning is an unexpected observation. This is because outcoupling is expected to decrease with increasing excimer-photonic mode energy separation. The observed emission can therefore not be readily explained from an optical perspective; a change in the excited state relaxation pathways is needed.

To get a deeper insight into how the excimer state is populated, excitation spectra were measured for the excimer emission at 520 nm (Figure 3b). Almost no population of the excimer states takes place when exciting the lower polariton of the most red-detuned cavity. The population mainly comes from the upper or middle polariton, both having quite high excitonic contributions. Here, prompt polariton emission when exciting the lower polariton can probably be viewed as close to elastic scattering, then preserving the photon momentum from the excited light and occurring too fast for any competing relaxation routes. It is therefore not visible in our measurement geometry. Comparing the normalized absorption and excitation spectra of the red-detuned cavity shows that the absorption of the lower polariton is quite high, whereas the excitation intensity is extremely low. In essence, the absorption and excitation spectra do not match. The difference between the excitation spectra and the absorption becomes smaller with decreased red-detuning and is almost the same for the most blue-detuned cavity. The same behavior is seen in 3,5-isopropyl-BODIPY cavities (Figure S5), so the phenomenon is not related to 1-ethyl-perylene specifically. The discrepancy between absorption and excitation spectra of strongly coupled systems has previously been observed.^49^ It therefore seems to be a general feature for molecular strong exciton–photon coupling.

To explain these observations, we need to consider different pathways. The transition from the lower polariton to the excimer can either be direct or via the exciton reservoir. The same is true for the transition from the excimer to the lower polariton. The reduced intensity of the lower polariton in the excitation spectra for the red-detuned cavity indicates that the transition efficiency to the excimer lowers with red-detuning. Three aspects could explain this. First, the driving force for a direct transition to the excimer is reduced with red-detuning. Second, the polariton-bare molecule energy overlap is reduced, lowering the rate of the pathway via the exciton reservoir. Third, only in the cavities with negligible polariton-bare molecule energy overlap is the polariton ideal, which is of importance for exciton–polariton dynamics. It is difficult to decipher which aspect that is of most importance. It is further difficult to draw any conclusions regarding any eventual transition from the excimeric state to the lower polariton. However, it is clear that excimer emission can be controlled by tuning the energetics of the polaritonic system.

### Delayed Emission Characteristics

**Figure 4:**
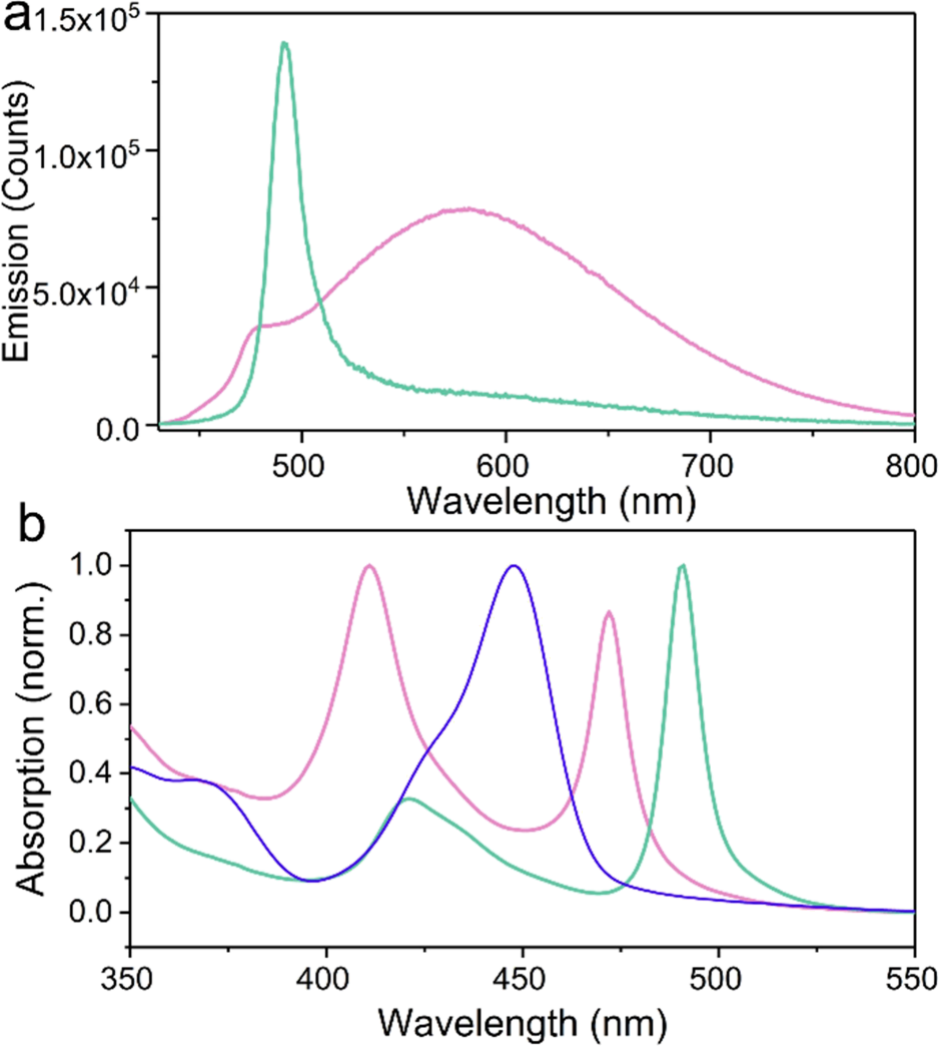
(a) Delayed emission spectra of the red-detuned (pink) and blue-detuned (green) cavities containing DABNA-2 after 50 ns and (b) normalized absorption spectra of the cavities in comparison with a pristine DABNA-2 film (blue).

After finding that the relaxation toward aggregation-induced trap states can be controlled in molecules having prompt emission by tuning the cavity energy, we will now turn our attention toward molecules exhibiting TADF. In the process of TADF, the excited singlet state is populated by RISC from the triplet state. Delayed emission is therefore seen for this kind of molecule. Having such a system in the strong coupling regime, the delayed emission (Figure 4) shows a similar tuning dependency to when using prompt emissive dyes. The blue-detuned cavity shows a higher degree of excimer emission, whereas the red-detuned cavity shows a much higher degree of polaritonic emission. Furthermore, the red-detuned cavity shows no dependency of the relative polaritonic emission with time after excitation (Figure S6a). However, for the blue-detuned cavity, the relative polaritonic emission lowers with time and at the same time is the excimeric emission slightly red-shifted (Figure S6b). This supports the previously discussed concept of trap states already formed on the triplet surface. Furthermore, with time deeper trap states are formed (seen from the red-shifted excimer emission), which have a lower tendency to relax through the lower polaritonic branch in the blue-detuned cavity.

It is tempting to use the same argumentation as to phenomenologically explain the delayed emission photophysics as for the prompt emission examples. However, the formation of excimers is different, which needs to be taken into account. Here, we know that we start with an excimeric state, the reduced excimeric emission in the strong coupling regime can therefore not be due to an avoided excimer formation. Instead, an excimer-to-polaritonic route must be present. The red-detuned cavity has a considerable lower energy compared to the blue-detuned one; it is further spectrally well separated from the bare molecule transition, reducing possible transitions from the polariton to the exciton reservoir. The energies of the lower polariton and the exciton reservoir are very similar. Small differences in polariton energy can therefore significantly change the energetic driving force for excimer to polariton transitions. Furthermore, if the lower polaritonic state is reached, radiative decay toward the ground state is rapid.

For the prompt emission discussed above, it could not be concluded if the reduced excimer emission in the strong coupling regime was a consequence of the rate of relaxation toward the polaritonic branch being faster as compared to the excimeric branch, or due to a direct excimer-to-polariton transition. Here, we start with an excimer; thus the absence of excimer emission in the strong coupling regime strongly indicates a direct excimer to polariton transition.

## Conclusions

From the experimental evidence presented here, it is clear that the strong coupling regime can mitigate the effect of aggregation-induced trap states formed in organic materials. When low-energy polaritonic states are present, aggregation-induced emission is reduced in favor of high-intensity and energetic polariton emission. However, the picture is not entirely clear on the mechanism of the dynamics between polaritonic and aggregational trap states. There are several mechanistic possibilities why the transition probability from polaritonic to trap states is reduced when the energy of the polariton lowers. Furthermore, our prompt emission data cannot distinguish between kinetic and thermodynamic effects. Thus, the removal of aggregation-induced prompt emission either can be due to the excited state energy relaxing slower to the trap state compared to the polariton, from where emission occurs faster than other deactivation pathways, or, a direct transition between the close to isoenergetic trap and polariton states. The comparative results obtained in three different molecules do however indicate that the effect observed here is general. The picture becomes clearer when observing delayed emission. Here, a trap state is first formed on the triplet manifold, from where it can reach a trap state on the singlet manifold. The energy now originates from an aggregational state; thus the removal of aggregation-induced emission indicates a direct transition from the aggregational state (either from the triplet or singlet manifold) to the polaritonic state. Close to isoenergetic energies between these surfaces probably facilitate such transitions. These findings shed light on the dynamics between polaritonic and aggregation-induced states, and the ideas presented here can mitigate issues with aggregation-induced emission in organic molecule-based photonic applications.

## Supporting Information Available

Absorption and emission spectra of PTCDA and 3,5-isopropyl-BODIPY; fitting result-coupled oscillator model; Hopfield coefficients; emission spectra of 1-ethyl-perylene cavities and the PTCDA cavity; emission lifetime decays of the 1-ethyl-perylene cavity and PTCDA cavity compared with the bare film; fitting results of lifetime decays; emission spectra using the cavity filter effect; emission and excitation spectra of the 3,5-isopropyl-BODIPY cavity and bare film; and time-resolved delayed emission spectra for DABNA-2 cavities (PDF)
