# Supplementary material for: Interplay between Polaritonic and Molecular Trap States
Source: J Phys Chem C Nanomater Interfaces. 2022 May 3;126(18):7965–72. doi: 10.1021/acs.jpcc.2c01239 (PMC9109220; doi:10.1021/acs.jpcc.2c01239)
Supplement: Supplementary file 1 — jp2c01239_si_001.pdf [file jp2c01239_si_001.pdf]

Supporting Information: “Interplay Between Polaritonic and Molecular Trap States”

*Jürgen Mony‡, Yi Yu‡, Clara Schäfer, Suman Mallick, Khushbu Kushwaha, and Karl Börjesson**

Department of Chemistry and Molecular Biology, University of Gothenburg, Kemigården 4, 41296 Gothenburg, Sweden

Additional Figures and Tables

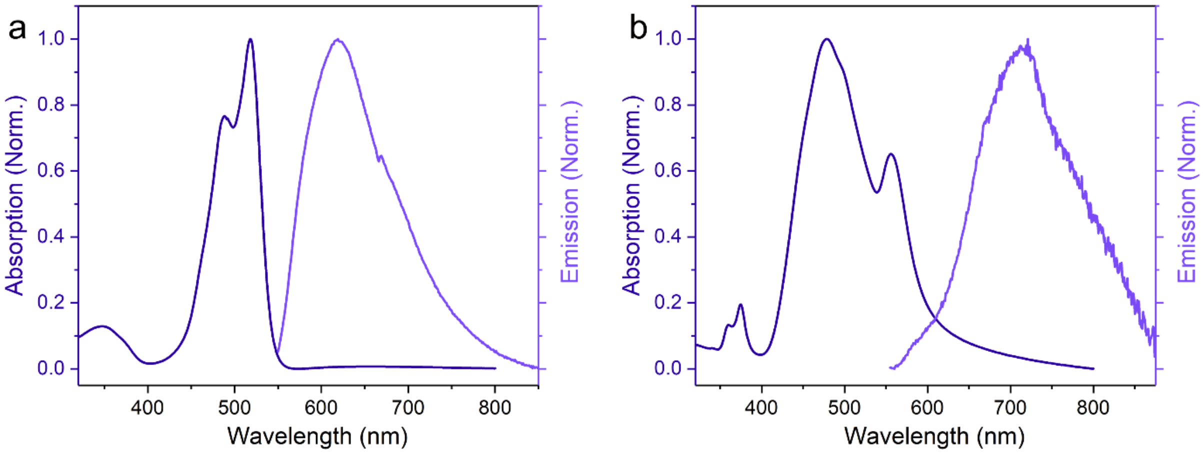

Figure S1: Absorption and emission spectrum of a) 3,5-isopropyl-BODIPY and b) perylenetetracarboxylic dianhydride (PTCDA).

Table S1: Fitting results from a three coupled harmonic oscillators model to 1-ethyl-perylene samples in Figure 2 with the energy of the cavity resonance $E_{Cav}(\theta)$ at the angle θ of 0 degree, the energy of the two excitonic transition E_{Exc} and the Rabi splitting $\hbar\Omega_R$.

	$\boldsymbol{E_{Cav}(\theta)}$ [eV]	$\boldsymbol{E_{Exc,1}}$ [eV]	$\boldsymbol{E_{Exc,2}}$ [eV]	$\hbar\boldsymbol{\Omega_{R,1}}$ [meV]	$\hbar\boldsymbol{\Omega_{R,2}}$ [meV]
Cavity a	2.72±0.02	2.82	3.00	348±15	384±15
Cavity b	2.86±0.03	2.82	3.00	344±14	376±14
Cavity c	2.98±0.02	2.82	3.00	338±10	370±10
Cavity d	3.14±0.02	2.82	3.00	332±10	374±10

Table S2: Hopfield coefficients $|\alpha|^2$, $|\beta|^2$ and $|\gamma|^2$ for the 1-ethyl-perylene containing cavities determined by the coupled harmonic oscillator model.

	$\boldsymbol{|\alpha|^2}$	$\boldsymbol{|\beta|^2}$	$\boldsymbol{|\gamma|^2}$
Cavity a	0.65±0.01	0.24±0.01	0.11±0.01
Cavity b	0.50±0.01	0.37±0.01	0.12±0.01
Cavity c	0.37±0.01	0.51±0.01	0.12±0.01
Cavity d	0.22±0.01	0.67±0.01	0.10±0.01

Table S3: Fitting results from a two coupled harmonic oscillators model to the DABNA-2 samples in Figure 2 with the energy of the cavity resonance $E_{Cav}(\theta)$ at the angle θ of 0 degree, the energy of the excitonic transition E_{Exc} and the Rabi splitting $\hbar\Omega_R$.

	$\boldsymbol{E_{Cav}(\theta)}$ [eV]	$\boldsymbol{E_{Exc}}$ [eV]	$\hbar\boldsymbol{\Omega_R}$ [meV]
Cavity e	2.68±0.02	2.79	420±6
Cavity f	2.85±0.03	2.79	400±7

Table S4: Hopfield coefficients $|\alpha|^2$ and $|\beta|^2$ for the DABNA-2 containing cavities determined by the coupled harmonic oscillator model.

	$\boldsymbol{|\alpha|^2}$	$\boldsymbol{|\beta|^2}$
Cavity e	0.63±0.01	0.37±0.01
Cavity f	0.43±0.01	0.57±0.01

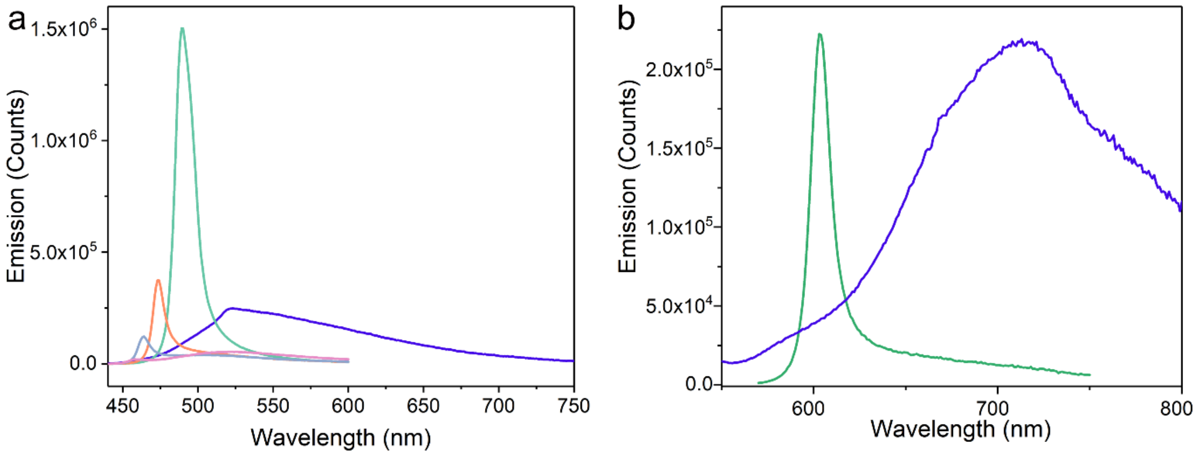

Figure S2: a) Emission spectra of 1-ethyl-perylene cavities with different tuning (green, orange, blue, pink) in comparison to the bare film emission (dark blue). b) Emission spectra of a PTCDA cavity (green) and a PTCDA solid film (dark blue). The same setup and measurement parameters were used for both measurements.

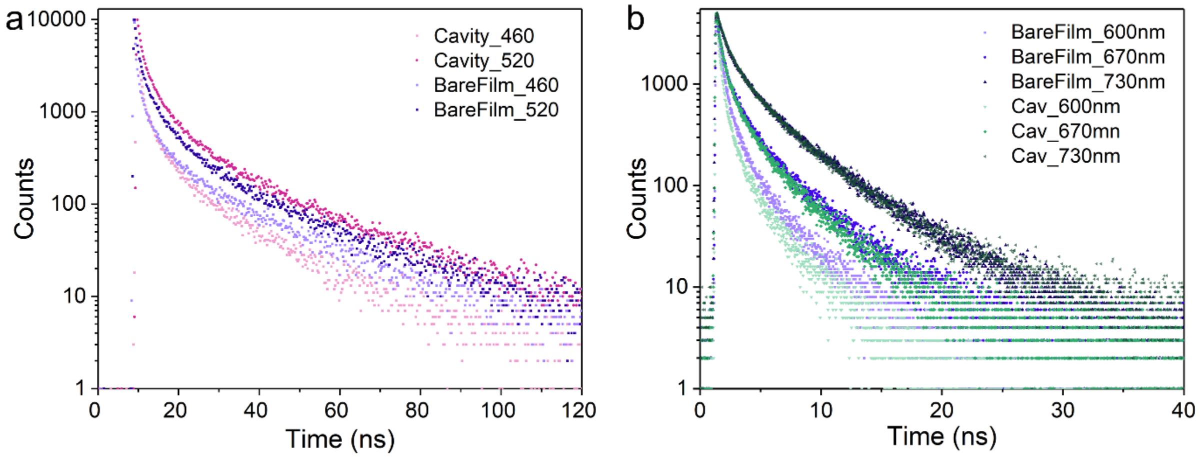

Figure S3: a) Emission lifetime measurements of the blue-detuned 1-ethyl-perylene cavity (pink) and the bare film (blue). The lighter spots are measured at a wavelength of 460 nm and the darker spots at a wavelength of 520 nm. Therefore, the lighter spots represents the decays of the polaritonic emission of the cavity or the molecular emission of the bare film and the darker spots the decays for the excimer emission for both samples. The excitation wavelength was 405 nm for both samples. b) Emission lifetime measurement of the PTCDA cavity (green) and the PTCDA solid film (blue). The decays were measured at the wavelength of the polaritonic/molecular emission at 600 nm (lighter dots), of the charge transfer states at 670 nm (medium dots) and of the excimer emission at 730 nm (darker dots).

Table S5: Pre-exponential factors (B) and lifetimes (τ) obtained from the reconvolution fittings of the emission decays for the blue-detuned 1-et-perylene cavity and the bare film displayed in Figure S3a. Errors are given as one standard deviation.

Decay	τ_1 (ns)	B_1	τ_2 (ns)	B_2	τ_3 (ns)	B_3
BareFilm 460	0.144±0.003	1.648±0.182	2.360±0.037	0.168±0.003	20.351±0.201	0.022±0.001
BareFilm 520	0.143±0.002	1.416±0.017	2.971±0.031	0.268±0.003	20.8361±0.173	0.035±0.001
Cavity 460	0.064±0.004	1.770±0.022	2.050±0.034	0.159±0.003	19.729±0.003	0.021±0.001
Cavity 520	0.367±0.003	0.810±0.014	4.450±0.003	0.269±0.003	25.645±0.186	0.049±0.001

Table S6: Pre-exponential factors (B) and lifetimes (τ) obtained from the reconvolution fittings of the of the emission decays for the PTCDA cavity and the PTCDA solid film displayed in Figure S3b. Errors are given as one standard deviation.

Decay	τ_1 (ns)	B_1	τ_2 (ns)	B_2	τ_3 (ns)	B_3
BareFilm 600	0.080±0.002	0.266±0.006	0.789±0.014	0.038±0.001	3.663±0.055	0.005±0.001
BareFilm 670	0.081±0.003	0.266±0.008	1.046±0.017	0.042±0.001	4.422±0.045	0.010±0.001
BareFilm 730	0.137±0.006	0.110±0.005	1.134±0.010	0.065±0.001	4.965±0.019	0.026±0.001
Cav 600	0.049±0.002	0.384±0.013	0.644±0.015	0.029±0.001	2.944±0.046	0.005±0.001
Cav 670	0.113 ±0.003	0.204±0.004	1.065±0.018	0.043±0.001	4.301±0.052	0.009±0.001
Cav 730	0.164±0.003	0.147±0.003	1.600±0.023	0.058±0.001	5.620±0.054	0.018±0.001

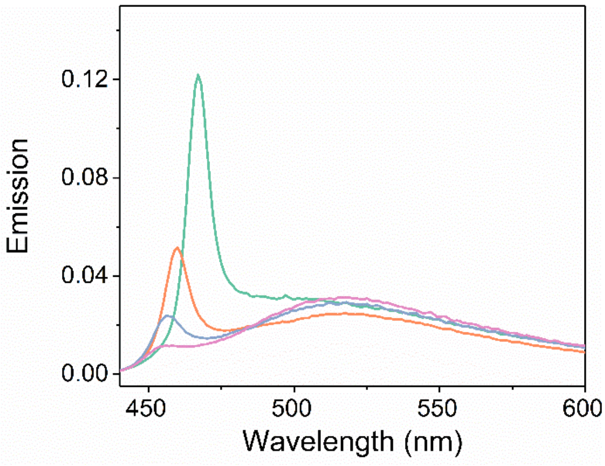

Figure S4: Emission spectra for the differently tuned 1-ethyl perylene cavities obtained by using the bare film emission filter by the reflectance of the cavities.

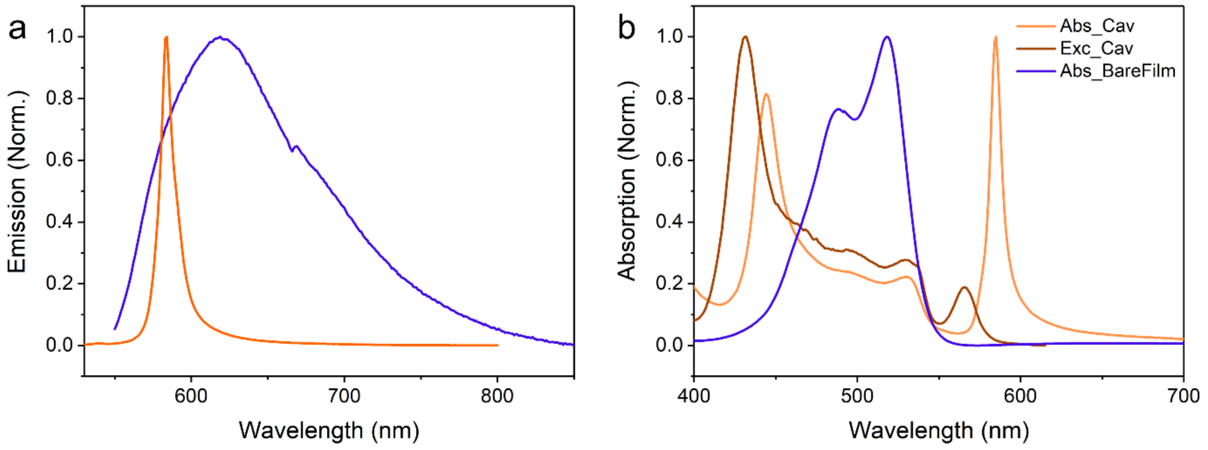

Figure S5: a) Normalized emission spectra of the 3,5-isopropyl-BODIPY cavity (orange) and the bare film (dark blue). b) Normalized absorption spectra of the 3,5-isopropyl-BODIPY cavity (bright orange) and the bare film (dark blue) in comparison with the excitation spectrum of the 3,5-isopropyl-BODIPY cavity (dark orange) for the excimer emission at a wavelength of 625 nm.

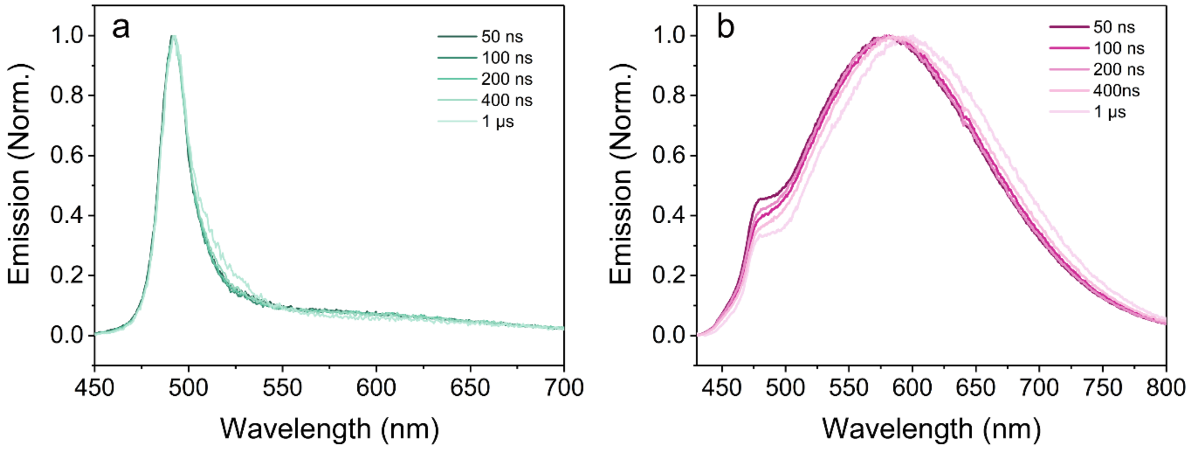

Figure S6: Normalized time resolved delayed emission spectra for the a) red-detuned and b) blue-detuned DABNA-2 containing cavities.
